# Research trends in farmers’ mental health: A scoping review of mental health outcomes and interventions among farming populations worldwide

**DOI:** 10.1371/journal.pone.0225661

**Published:** 2019-12-05

**Authors:** Briana N. M. Hagen, Ashley Albright, Jan Sargeant, Charlotte B. Winder, Sherilee L. Harper, Terri L. O’Sullivan, Andria Jones-Bitton

**Affiliations:** 1 Department of Population Medicine, Ontario Veterinary College, University of Guelph, Guelph, Ontario, Canada; 2 Centre for Public Health and Zoonoses, University of Guelph, Guelph, Ontario, Canada; 3 School of Public Health, University of Alberta, Edmonton, Alberta, Canada; University of Milan, ITALY

## Abstract

**Background:**

Mental health issues among farmers are identified population health concerns. While one systematic review focused on suicide in farming populations in the United States, there have been no scoping studies examining mental health in farming communities worldwide. The objectives of this scoping review were to: provide a descriptive analysis of the literature pertaining to mental health outcomes in farming populations; describe the international scope of the research; and highlight published mental health services and interventions that have been evaluated.

**Methods:**

Following Arksey and O’Malley’s scoping review framework, five major health and science platforms were used to identify studies examining mental health outcomes in farming populations, worldwide. Studies that met *a priori* inclusion criteria that were published prior to December 31, 2017 were included in this review. Data synthesis and descriptive statistics were conducted using STATA 15® software; proportions were calculated by country.

**Results:**

The initial literature search yielded 9,906 records. After title and abstract screening, 676 articles were reviewed in-full. Of these, 341 met *a priori* inclusion criteria. Studies included were conducted between 1979 and 2017; the majority (265; 77.7%) were published between 2002–2017. The most frequently measured outcomes were stress (41.9%), suicide (33.1%), and depression (32.6%). Over 70% of studies that examined stress described using quantitative research methods, most predominantly, cross-sectional designs (42.7%). Approximately 64% of studies that measured suicide reported using a quantitative approach; the largest proportion of included suicide studies (33.6%) described using cohort designs. Approximately 84% of studies that measured depression described using quantitative approaches; sixty percent of these studies reported using a cross-sectional study design. Twenty included studies described a mental health service or intervention (5.9%).

**Conclusions:**

This scoping review provides a critical overview of the literature examining mental health outcomes in farming populations worldwide. Given the importance of farming and agriculture, this review can be used to ensure future research complements existing work, avoids unnecessary overlap, and begins to tackle the less-studied mental health outcomes amongst farmers. These results can guide researchers to identified gaps in research and services, leading to a more informed approach to future work, and ultimately, a more comprehensive understanding of mental health among farmers worldwide.

## Introduction

Farmer mental health can have impacts on individual health [1–3], family life [4], [5], farm productivity [6], and animal health and welfare [7]. Considering that approximately one-third of individuals that contribute to the global economy through their employment do so through the agricultural industry [8], poor mental health could therefore have considerable negative impact on economic productivity, animal health, and human health worldwide. Hence, ensuring the mental health of farmers and farmworkers may be essential for global health.

It is estimated that 1 in 4 people worldwide experience issues with their mental health annually [9]. If farmers experience problems with their mental health at the same rates as the general population, this would mean that approximately 25% of farmers worldwide are struggling with their mental health every year. Globally, there are more than 570 million farms, of which approximately 550 million are family-run [8]. With a conservative estimate of 2-member families, this would mean that every year, roughly 225 million farmers worldwide may struggle with their mental health. This number is likely an underestimate when considering the evidence that farmers experience mental illness at a higher rate than the general population [10], coupled with the fact that farmworkers and other farm helpers are not included within that conservative estimate.

One recent systematic review conducted by Klingleschmidt and colleagues (2017) reported that male farmers in the United States of America (US) experience an increased risk of suicide (pooled effect size = 1.48; 95% CI: 1.30–1.68) compared to the general population [11]. Additionally, it is well established through psychological autopsy studies conducted in the US and Europe that over 90% of people who died by suicide had experienced issues with their mental health [12]. It is essential that mental illness and associated risk factors be assessed in farming populations. Highlighting which mental health outcomes and associated risk factors have previously been studied and, more specifically, how they are studied among farming populations, can inform future research and mental health intervention planning. To date, there is no comprehensive review of mental health outcomes among farming populations worldwide or any that explore mental health interventions for farming populations.

Across the globe, farmers, community groups, mental health experts, and governmental agencies have called for making farmer mental health a research and policy priority [8]. While there are a multitude of individual studies examining mental health outcomes in farming populations across the world, there are no methodological guidelines for measuring individual mental health outcomes in these populations. Such guidelines could help researchers compare findings across countries and build on previous work more efficiently.

In order to determine the breadth of the existing research and identify current gaps in knowledge, we conducted a scoping review of the literature. Scoping reviews map the existing literature, especially in areas that are rapidly evolving [13], such as farmer mental health. The objectives of the scoping review were to: (a) provide a descriptive analysis of the literature pertaining to mental health outcomes in farming populations; (b) describe the international scope of the research; and (c) highlight the mental health services and interventions that have been evaluated in the literature to help identify successes and limitations for implementing services in farming populations.

## Methods

Following the guidelines set out by Arksey and O’Malley (2005) and reported according to the Preferred Reporting Items for Systematic Reviews and Meta-Analysis extension for scoping reviews (PRISMA-ScR) guidelines [14], this scoping review was designed with an analytical framework that maps the available literature, describing the study types and outcomes assessed.

### Protocol and registration

Prior to conducting the search, we developed our review question, eligibility criteria, screening questions, and data extraction tool (S1 Appendix). There was no formal registration of a study protocol with the international systematic review database (PROSPERO).

### Eligibility criteria

This review was designed to be broad in scope and range to describe the nature and extent of the literature investigating aspects of mental health among farming populations. Eligibility criteria for inclusion in the review were the following:

- The study population explicitly included farmers or agricultural workers (from any farming commodity)
- The full text of the research article was accessible in English
- The article was a primary study, a review, or governmental report
- If a primary study, studies that used primary data collection (i.e. the authors collected the data themselves in order to answer their research question) or secondary data analysis (i.e. the authors did not collect the data themselves but used an existing dataset or census data)
- The outcome(s) investigated were mental health outcome(s). This could include negative (e.g. depression, anxiety) or positive (e.g. resilience) mental health outcomes.

### Information sources and electronic search

Databases searched were: Agricola (via ProQuest, 1970 to current), CABI (via CAB Direct, 1973 to current), PubMed (via NCBI, 1950 to current), Science Citation Index Expanded (via Web of Science, 1900 to current), Social Sciences Citation Index (via Web of Science, 1900 to current), Arts & Humanities Citation Index (via Web of Science, 1975 to current), Conference Proceedings Citation Index-Science (via Web of Science, 1990 to current), Conference Proceedings Citation Index–Social Science & Humanities (via Web of Science, 1990 to current), Emerging Sources Citation Index (via Web of Science, 2015 to current), Medline (via Ovid, 1950 to current), and PsycINFO. These platforms were chosen based on the breadth of their journal libraries in the databases and use in mental health scoping reviews in other populations [15–19].

The search strategy was developed in consultation with a library scientist at the University of Guelph to include the following concepts: mental health outcomes or mental wellness, and farming populations. The full search string is available in Table 1. We chose to use the terms “clinical depression” and “depressive symptom” to capture studies measuring depression, as the term “depression” was considered too broad. To verify these terms were adequately sensitive, key studies were pre-selected by the primary author to ensure they were captured in the search. Additionally, studies examining positive mental health outcomes typically have the term “resilience” nested within the concept of “mental wellbeing”. When resilience was tested as an additional search term, no additional relevant records were identified; therefore, this term was removed for parsimony. One author (AA) executed the initial search strategy and a second author (BH) replicated the search. There were no timeline restrictions. The initial search was conducted in July 2017 and a second search was conducted using the same methodology in April 2018 to capture studies published in press or online before December 31, 2017.

**Table 1:** Search terms used to perform scoping review literature search in July 2017 and April 2018 in all databases, including Agricola, CAB Direct, PubMed, Web of Science, and PsychInfo.

Database	Search string
Master search string	(“Mental health” OR “Mental health services” OR “Mental illness” OR “mental wellbeing” OR anxiety OR “clinical depression” OR “occupational stress” OR suicide* OR “depressive symptom*”) AND (farm* OR agricultur*)

An *a priori* decision was made to examine reference lists for all review articles after full-text screening to identify any potentially relevant articles not found in the electronic search. These records were then screened at the title and abstract phase for inclusion.

### Study selection

The records identified by the search were exported from the platforms into Mendeley^TM^ where duplicate articles were removed. Next, titles and abstracts were screened for eligibility for inclusion in the full-text screening. Titles and abstracts were screened independently by two authors (AA and BH) using the following questions:

1. Does the study examine a mental health outcome as a primary outcome of interest?
2. Does the study examine a farming population?
3. Is the study available in English?

For each question, answers could have been “yes”, “no”, or “unclear”. Citations were excluded if the reviewers answered “no” to any of the screening questions. Any discrepancies in selection were discussed to achieve consensus. If consensus could not be reached, a third reviewer was used. Articles with ambiguous abstracts or abstracts that were not available online were deemed as eligible in this title and abstract phase and were evaluated using the full text.

After title and abstract screening, eligible full-text articles were imported into DistillerSR™. Full texts of all articles were retrieved through online databases or through the Rapid Access to Collections by Electronic Requesting (RACER). RACER includes all databases from all libraries that are members of the Ontario Council of University Libraries. Articles were excluded if they were inaccessible online or through RACER, or were academic theses or books.

### Data charting process

Four authors (BH, AA, JS, AJB) developed a comprehensive data extraction form, which was pre-tested by two authors (BH, AA) on ten articles to ensure consistency in form completion. The data extraction was completed in DistillerSR^TM^ by the same two authors for each article that met the inclusion criteria; any conflicts were discussed to achieve consensus (S1 Appendix).

### Data items

Data of interest in this review included: the country of study; year of publication; the mental health outcome(s) measured; farming population studied (farm level and individual level); the methodological approach to the data (qualitative, quantitative, mixed methods, or review); whether the study used primary or secondary data or was a review article; and the specific study design.

#### Study demographics

Farming populations of interest included: animal farmers (swine, beef, dairy, small ruminants (meat or dairy), poultry, aquaculture, or other); plant farmers (crops and/or horticulture); migrant farm workers; permanent farm workers; farm families; not specified; or other. These items were collected as a list, where more than one data item could be selected. Where the study examined a farming population that was not an option on the data extraction form, there was an open-text box where the commodity was entered manually (S1 Appendix). We defined migrant and permanent farmworkers as farmworkers who left their country of origin to find agricultural employment (seasonally and permanently, respectively). Further, individual level populations of interest were identified through the “other” text-box variable, and included female farmers, male farmers, older farmers, farm managers, and farm owners.

#### Study characteristics

Observational studies were categorized as descriptive, hypothesis testing, or both. Descriptive studies were reported as studies that estimated the prevalence of a mental health outcome, evaluated attitudes towards mental health outcomes, or both. Hypothesis testing studies were reported as studies that evaluated an intervention, investigated risk factors, or evaluated a diagnostic criterion. Qualitative study designs were reported as phenomenology, grounded theory, ethnographic, case study, biography, or other.

#### Mental health services and interventions

Interventions reported could include behaviour modification, health measures, efficacy, or other. Additional data extracted for these studies included funding information, whether the service was informational or interventional, who developed the service, who delivered the service, how the service was delivered, how the service was/is funded, and whether the service has been formally evaluated in the literature. Given the limited number of intervention studies found, in addition to the formal data extraction approach taken with the mental health outcomes, we reported a more in-depth description of the mental health services and interventions. This in-depth review was conducted by the primary author.

### Synthesis of results

Data synthesis and descriptive statistics (proportions) were conducted using STATA 15® data software; proportions were calculated by mental health outcome.

## Results

### Studies included

**Fig 1:**
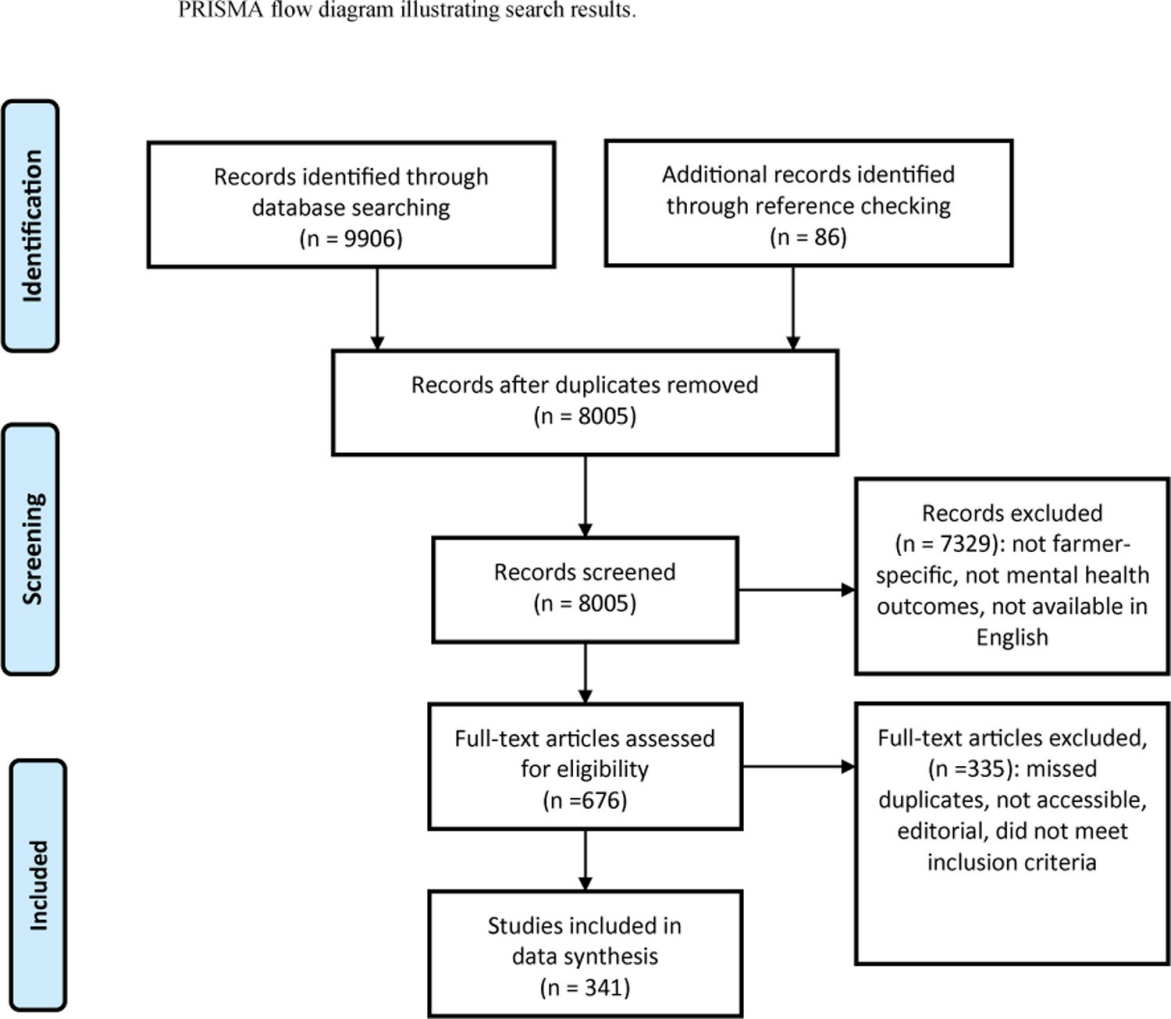
PRISMA flow diagram illustrating studies that were identified using the search strategy, articles that were screened for eligibility, included/excluded with reasons, and studies included in the data extraction (Moher et al., 2009).

The initial search conducted in July 2017 yielded a total of 9906 articles: 430 identified in Agricola, 2374 in CAB Direct, 2797 in Pubmed, 1516 in Web of Science, 494 in PsycINFO, and 2295 in Medline. Title and abstract screening resulted in 676 articles eligible for full review; of these, 341 articles were included in the data extraction (Fig 1).

### Overview of the data–study context

**Fig 2:**
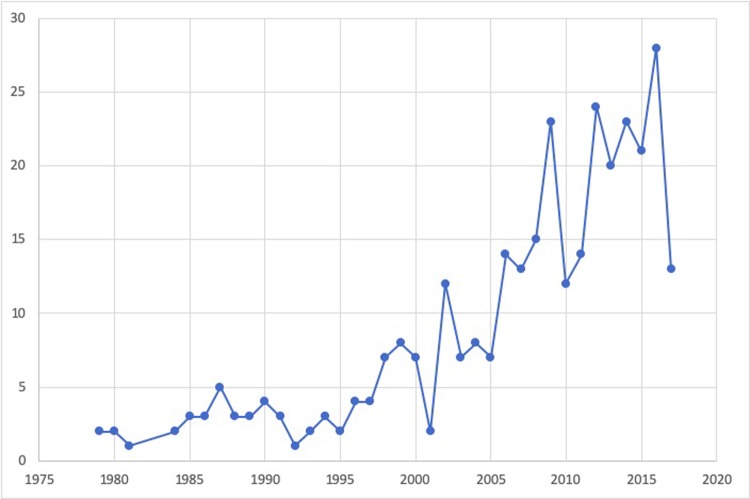
The number of studies examining a mental health outcome in a farming population by year of publication.

Studies were published between 1979–2017 (Fig 2). A large proportion of the literature was published since the year 2000 (80.9%), and 48.1% had been published between 2010 and 2017.

**Fig 3:**
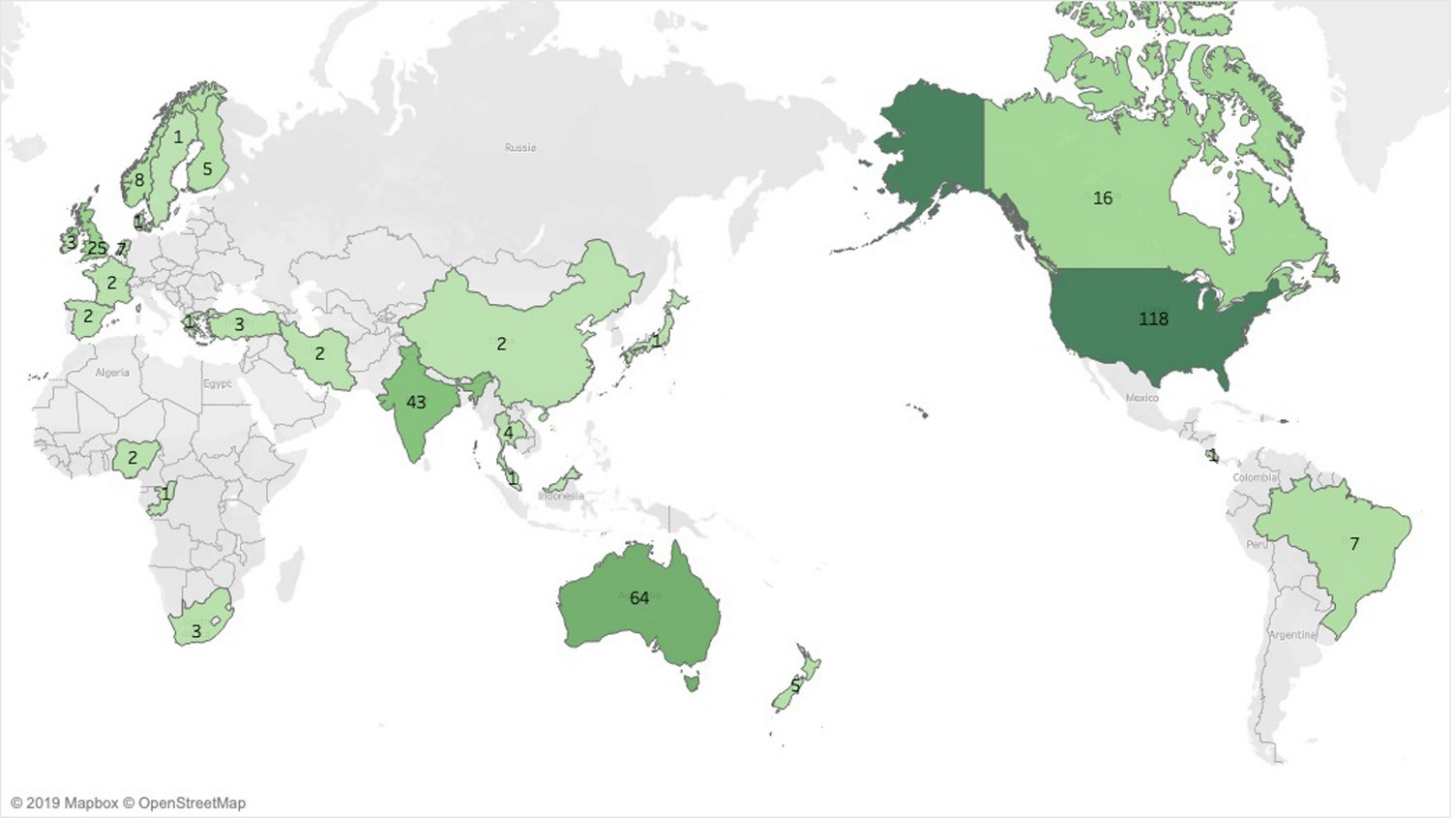
World map indicating where studies of mental health outcomes in farming populations were conducted (1979–2017).

Twenty-seven countries were represented in the literature (Fig 3). As shown in Table 2, the largest proportion of studies were conducted in the United States (34.6%), followed by Australia (18.8%), India (12.6%), and the United Kingdom (7.3%).

**Table 2:** Primary mental health outcome studied by country (n = 341).

Country	Total number of studies n (%)	Suicide n (%)	Stress n (%)	Depression n (%)	Anxiety n (%)	Resilience n (%)	Mortality n (%)	Burnout n (%)	Mental Health Service n (%)	Non-specified mental health outcome n (%)
United States	118 (34.6)	11 (9.7)	60 (41.9)	59 (53.2)	20 (39.2)	9 (45.0)	1 (14.3)	0	4 (20.0)	7 (28.0)
Australia	64 (18.8)	19 (16.8)	27 (18.9)	11 (9.9)	7 (13.7)	8 (40.0)	2 (28.6)	0	12 (60.0)	6 (24.0)
United Kingdom	25 (7.3)	9 (8.0)	12 (8.4)	8 (7.2)	6 (11.8)	0	0	0	1 (5.0)	3 (12.0)
India	43 (12.6)	42 (37.2)	3 (2.1)	2 (1.8)	1 (2.0)	0	1 (14.3)	0	1 (5.0)	0
Canada	16 (4.7)	5 (4.4)	11 (7.7)	2 (1.8)	2 (3.9)	2 (10.0)	0	1 (50.0)	1 (5.0)	1 (4.0)
Brazil	7 (2.1)	4 (3.5)	1 (0.7)	2 (1.8)	2 (3.9)	0	1 (14.3)	0	0	2 (8.0)
New Zealand	5 (1.5)	2 (1.8)	4 (2.8)	0	0	0	1 (14.3)	0	0	0
Finland	5 (1.5)	2 (1.8)	2 (1.4)	0	0	0	0	1 (50.0)	0	1 (4.0)
Netherlands	7 (2.1)	0	6 (4.2)	1 (0.9)	0	0	0	0	0	0
Norway	8 (2.4)	0	2 (1.4)	6 (5.4)	5 (9.8)	0	1 (14.3)	0	0	1 (4.0)
Thailand	4 (1.2)	0	3 (2.1)	1 (0.9)	0	0	0	0	0	1 (4.0)
South Korea	4 (1.2)	2 (1.8)	0	2 (1.8)	0	0	0	0	0	0
Turkey	3 (0.9)	0	1 (0.7)	1 (0.9)	0	0	0	0	0	1 (4.0)
China	2 (0.6)	1 (0.9)	1 (0.7)	1 (0.9)	1 (2.0)	0	0	0	0	0
France	2 (0.6)	1 (0.9)	0	1 (0.9)	0	0	0	0	0	0
Greece	1 (0.3)	0	0	1 (0.9)	0	0	0	0	0	0
Iran	2 (0.6)	0	1 (0.7)	2 (1.8)	2 (3.9)	0	0	0	0	0
Ireland	3 (0.9)	1 (0.9)	1 (0.7)	1 (0.9)	1 (2.0)	0	0	0	0	0
Japan	1 (0.3)	0	1 (0.7)	0	0	0	0	0	0	0
Congo	1 (0.3)	0	1 (0.7)	1 (0.9)	0	0	0	0	1 (5.0)	0
Denmark	1 (0.3)	0	0	0	0	0	0	0	0	1 (4.0)
Malaysia	1 (0.3)	0	1 (0.7)	1 (0.9)	1 (2.0)	0	0	0	0	0
Nigeria	2 (0.6)	0	1 (0.7)	1 (0.9)	1 (2.0)	0	0	0	0	0
South Africa	3 (0.9)	3 (2.7)	0	1 (0.9)	0	0	0	0	0	0
Sweden	1 (0.3)	0	0	0	0	0	0	0	0	1 (4.0)
Spain	2 (0.6)	2 (1.8)	0	0	0	0	0	0	0	0
Costa Rica	1 (0.3)	1 (0.9)	1 (0.7)	1 (0.9)	1 (2.0)	0	0	0	0	0
International	9 (2.6)	8 (7.1)	3 (2.1)	5 (0.9)	1 (2.0)	1 (5.0)	0	0	0	0
**Total**	**341(100.0)**	**113 (100.0)**	**143 (100.0)**	**111 (100.0)**	**51 (100.0)**	**20 (100.0)**	**7 (100.0)**	**2 (100.0)**	**20 (100.0)**	**25 (100.0)**

### Outcomes of interest

Overall, 75.1% of the studies collected primary data to examine mental health outcome(s), while 18.5% used secondary data sources, such as data collected through census surveys or vital statistics (S1 Table); review articles and governmental reports accounted for 6.5% of the included studies. The majority of all studies used quantitative research methods (72.1%); qualitative research methods and mixed methods represented 14.4% and 7.0% of studies, respectively.

### Mental health outcomes

Stress was the most prevalent outcome of interest, accounting for 41.9% (n = 143) of all studies (Table 2). The second most common outcome was suicide (33.1% of all studies; 111/341), followed closely by depression (32.6% of all studies). Anxiety was an outcome of interest in 15.0% of studies worldwide (51/341). Resilience was studied in 5.9% (20/341) of all studies.

#### Stress (n = 143)

Studies that examined stress as a mental health outcome of interest were represented across the full range of publication years (1979–2017). As shown in Table 2, four countries contributed over 75% of the literature around stress among farmers: US (41.9%), Australia (18.9%), United Kingdom (UK) (8.4)%, and Canada (7.7%). As shown in Table 3, over 70% of this research was quantitative in nature, with 42.7% of studies using a cross-sectional study design, followed by cohort studies, which accounted for 21.0% of the included studies. Approximately 15% of studies examining stress were qualitative and 7.7% were mixed methods studies; twenty-five of these studies reported using a phenomenological approach and two studies used an ethnographic approach.

**Table 3:** A comparison of mental health research conducted in farming populations worldwide (n = 341). Studies may have reported on more than one mental health outcome.

		Suicide	Stress	Depression	Anxiety	Resilience	Mortality	Burnout	Mental Health Service	Non-specified mental health outcome
**Publication range**		1990–2017	1979–2017	1980–2107	1980–2017	1980–2017	2002–2016	2016–2017	1998–2016	1979–2017
**Number of studies**		113 (33.1)	143 (41.9)	111 (32.6)	51 (15.0)	20 (5.9)	7 (2.1)	2 (0.6)	20 (5.9)	25 (7.3)
**Population**										
**Farm type**	*Animal farming*	1 (0.9)	37 (25.9)	9 (8.1)	7 (13.7)	5 (25.0)	0	1 (50.0)	2 (10.0)	4 (16.0)
	*Crop/Horticultue farming*	14 (12.4)	25 (17.5)	12 (10.8)	6 (11.8)	3 (15.0)	2 (28.6)	0	0	1 (4.0)
	*Both*	9 (8.0)	14 (10.0)	6 (5.4)	3 (5.9)	0	1 (14.3)	0	0	2 (8.0)
	*No reported farm type*	89 (78.8)	67 (46.9)	84 (75.7)	35 (68.6)	12 (60.0)	4 (57.1)	1 (50.0)	18 (90.0)	18 (72.0)
**Individual level**^**1**^										
	*Farmer*: *dairy*	9 (8.0)	26 (18.2)	9 (8.1)	4 (7.8)	3 (15.0)	0	1 (50.0)	1 (5.0)	4 (16.0)
	*Farmers*: *beef*	6 (5.3)	14 (9.8)	7 (6.3)	3 (5.9)	2 (10.0)	0	0	0	2 (8.0)
	*Farmer*: *swine*	4 (3.5)	7 (4.9)	1 (0.9)	0	0	0	0	1 (5.0)	4 (16.0)
	*Farmer*: *small ruminants*	8 (7.1)	11 (7.7)	3 (2.7)	0	1 (5.0)	0	0	0	2 (8.0)
	*Farmer*: *poultry*	5 (4.4)	5 (3.5)	2 (1.8)	1 (2.0)	0	0	0	0	3 (12.0)
	*Farmer*: *aquaculture*	2 (1.8)	2 (1.4)	1 (0.9)	0	0	0	0	0	0
	*Farmer*: *crops*	23 (20.4)	21 (14.7)	14 (12.6)	6 (11.8)	3 (15.0)	3 (42.9)	0	0	3 (16.0)
	*Farmer*: *horticulture*	8 (7.1)	11 (7.7)	6 (5.4)	3 (5.9)	2 (10.0)	0	0	0	0
	*Farmer*: *other commodity*^*2*^	3 (2.7)	0 (0.0)	1 (0.9)	1 (2.0)	0	0	0	0	1 (4.00)
	*Farm families*	7 (6.2)	36 (25.2)	18 (16.2)	5 (9.8)	8 (40.0)	1 (14.3)	0	8 (40.0)	3 (12.0)
	*Migrant farm workers*	2 (1.8)	28 (19.6)	29 (26.1)	15 (29.4)	1 (5.0)	0	0	1 (5.0)	4 (16.0)
	*Permanent farm workers*	17 (15.0)	12 (8.4)	10 (9.0)	4 (7.8)	0	3 (42.9)	0	2 (10.0)	2 (8.0)
	*Female farmers*	2 (1.8)	7 (4.9)	10 (9.0)	5 (9.8)	2 (10.0)	0	0	0	0
	*Male farmers*	6 (5.3)	2 (1.4)	5 (4.5)	2 (3.9)	0	0	0	1 (5.0)	0
**Study Type**										
	*Quantitative*	72 (63.7)	101 (70.6)	93 (83.7)	43 (84.3)	12 (60.0)	3 (42.9)	2 (100.0)	8 (40.0)	16 (80.0)
	*Qualitative*	20 (17.7)	22 (15.4)	4 (3.6)	3 (5.9)	4 (20.0)	1 (14.3)	0	3 (15.0)	5 (20.0)
	*Mixed methods*	5 (4.4)	11(7.7)	5 (4.5)	2 (3.9)	2 (10.0)	1 (14.3)	0	5 (25.0)	3 (12.0)
	*Review/governmental report*	16 (14.2)	9 (6.3)	9 (8.1)	3 (5.9)	2 (10.0)	2 (28.6)	0	4 (20.0)	1 (4.0)
**Study Design**										
	*Cross-sectional*	26 (23.0)	61 (42.7)	67 (60.4)	32 (62.8)	10 (50.0)	0	2 (100.0)	2 (10.0)	13 (52.0)
	*Case-control*	13 (11.5)	5 (3.5)	2 (1.8)	1 (2.0)	0	0	0	0	0
	*Cohort*	38 (33.6)	30 (21.0)	20 (18.0)	7 (13.7)	1 (5.0)	3 (42.9)	0	1 (5.0)	2 (8.0)
	*Quasi-experimental*	0	2 (1.4)	1 (0.9)	3 (5.9)		0	0	0	0
	*Randomized Control Trial*	0	0	2 (1.8)	1 (2.0)	0	0	0	2 (10.0)	0
	*Phenomenology*	10 (8.9)	25 (17.5)	8 (7.2)	3 (5.9)	6 (30.0)	2 (28.6)	0	2 (10.0)	7 (28.0)
	*Ethnography*		2 (1.4)	0	0	0	0	0	0	0
	*Case Study*	4 (3.5)	0	0	0	0	0	0	0	0
	*Service Evaluation*	1 (0.9)	2 (1.4)	1 (0.9)	1 (2.0)	0	0	0	7 (35.0)	0
	*Scale development/* *validity testing*	0	2 (1.4)	1 (0.9)	0	1 (5.0)	0	0	0	1 (4.0)
	*Theoretical framework development*	2 (1.8)	1 (0.7)	0	0	0	0	0	1 (5.0)	1 (4.0)
	*Literature Review*	19 (16.8)	13 (9.1)	9 (8.11)	3 (5.9)	2 (10.0)	2 (28.6)	0	5 (25.0)	1 (4.0)

Note: Studies could contribute to more than one farm type or individual level. Therefore, columns may not add to 100%.

^1^More than one individual level group could be investigated within one study. Therefore, numbers will not add to 100%.

^2^Other animal farmers included equine, livestock (unidentified), and worms (silk and compost).

#### Suicide (n = 113)

Suicide was reported as an outcome of interest in 33.1% (113/431) of included studies. As reported in Table 2, 16 individual countries contributed studies; over a third (37.2%) of suicide studies were conducted in India, followed by approximately 17% in Australia, 9.7% in the US, and 8% in the UK.

As reported in Table 3, nearly 64% of studies examining suicide were quantitative in nature; approximately 21% of these studies used cohort designs, 15% used cross-sectional designs, and 5.3% were case control studies. Almost 80% of studies did not specify a farming population of interest (78.8%) instead just referring to “farmers” generally. Of the studies that did identify a farming population, crop farmers were the most represented in studies that examined suicide (20.4%), followed by permanent farmworkers (15.0%).

#### Depression (n = 111)

Depression was reported as a mental health outcome of interest in 32.6% of included studies. Over half of the literature examining depression among farming populations was conducted in the US (53.2%). The next largest contributors were Australia, UK, and Norway, accounting for 9.9%, 7.2%, and 5.4% of the included studies, respectively.

As depicted in Table 3, 93/111 (83.7%) of the studies that examined depression as an outcome of interest were quantitative. The majority of depression studies reported using a cross-sectional approach (60.4%). Migrant and permanent farmworker populations were the most represented farming groups and were included in 36.6% of all included studies.

#### Anxiety (n = 51)

Fifteen percent of included studies examined anxiety as a mental health outcome of interest (51/341). The US contributed the largest proportion of studies examining anxiety (39.2%), followed by Australia (13.7%), UK (11.8%) and Norway (9.8%) (Table 2).

Quantitative research methods were reported in 84.3% of anxiety studies. These quantitative studies consisted largely of cross-sectional study designs (62.8%), while cohort study designs were reported in 13.7% of included studies. Quasi-experimental designs were reported in three studies examining anxiety (5.9%), and one randomized control trial was included (2.0%). Migrant and permanent farmworkers were the most prevalent study population of interest (37.2%), followed by farm families and female farmers (9.8% each). A large proportion of studies that examined anxiety did not report a farming population of interest (68.6%).

#### Resilience (n = 20)

Resilience was a mental health outcome of interest in 5.9% of the included studies (20/341) (Table 2). These studies were conducted in the US (45.0%), Australia (40.0%), Canada (10.0%), and one study that was international in scope (5.0%).

Sixty percent of studies that explored resilience used a quantitative approach including one cohort study (5.0%), one scale development/validity testing study (5.0%), and ten cross-sectional studies (50.0%). Forty percent of resilience studies reported farm families as their population of interest (n = 8), while 60% of studies had no reported farm type (Table 3). Resilience was the only positive mental health outcome observed in the search.

#### Mortality (n = 7)

Mortality studies that examined farmer mental health appeared in the literature in 2002 (Table 3). Since 2002, seven studies reported on mortality from mental health struggle, which was 2.1% of the mental health literature in farming populations. As depicted in Table 2, two of studies that reported mortality were conducted in Australia (28.6%), while India, US, New Zealand, and Norway each contributed one study.

Three mortality studies reported using quantitative methods, more specifically, a cohort study design (42.9%). Two studies reported using a phenomenological approach to qualitative data (28.6%) and two studies were literature reviews (28.6%). Four mortality studies did not report if they had a particular farming population of interest (57.1%). Among the studies that did report a population of interest, crop farmers and permanent farm workers were indicated in three studies (42.9%)

#### Burnout (n = 2)

Two studies reported examining burnout as a mental health outcome of interest among a farming population; one study was conducted in Canada and one in Finland. Both of these studies used a cross-sectional approach to estimate the prevalence of burnout among farming populations. The Canadian study did not specify a farming population of interest, while the Finnish study examined dairy farmers.

#### Non-specified mental health outcome (n = 25)

In 7.3% of studies, a specific mental health outcome was not identified, but rather, the broad construct of mental health or psychological wellness was examined. Eighty percent of these studies had a quantitative study type: 52.0% used a cross-sectional study design and 8.0% used a cohort study design to measure mental health. Qualitatively, 28.0% of studies used a phenomenological approach to exploring mental health among farmers.

#### Risk factors associated with mental health outcomes

As outlined in Table 4, examined risk factors related to farmer mental health included: demographic information (19.9%) and socioeconomic status (7.6%); help-seeking (6.2%); pesticide use (5.0%); substance use (5.0%); and climate change (2.3%). Physical health was represented in approximately 15.2% of all studies.

**Table 4:** Factors of interest explored among 341 studies included in data extraction.

Risk factors studied	*n*	Proportion of total (n = 341)
Demographics	68	19.9%
Physical health	52	15.2%
Socioeconomic status	26	7.6%
Help seeking	21	6.2%
Farm-related variables	20	5.9%
Farmer-related variables	18	5.3%
Farm family dynamics	18	5.3%
Pesticide use	17	5.0%
Substance use/abuse	17	5.0%
Coping	10	2.9%
Social support/community	10	2.9%
Climate	8	2.3%
Mental health support	7	2.1%
Legislation/political issues	3	0.9%
Cultures of masculinity	1	0.3%
Technology	1	0.3%
Culture	1	0.3%
Marginalization	1	0.3%

### Mental health interventions

Twenty studies described a mental health service or resource (5.9% of all studies). These were conducted between 1998 and 2016.

#### Australia (n = 12)

In Australia, twelve studies described or assessed mental health services (60.0%) [20]–[26]. Four of these studies focused on assessing the efficacy of increasing mental health literacy among farmers and those who work with farmers [21]–[23], [27]. One mixed-methods study, conducted by Brumby et al (2009), provided an overview of their approach to farmer physical and mental health through “The Sustainable Farm Families (SFF)” project. This comprehensive approach to health and wellness highlighted the need for focusing on the farm family as a unit and engaging families as active learners [22]. Using a workshop format, this intervention consisted of a two-day workshop, followed by a one-day workshop in years two and three of the three-year intervention. These workshops combined physical and mental health education, and both pre- and post-training data were collected by questionnaire to assess knowledge and understanding of the core topics covered. Results showed a statistically significant change in knowledge around participant’s basic understanding of rural health facts, disease processes (including risk factors), and lifestyle questions that was maintained over the 3-year project for both men (85% retention; p<0.05) and women (86% retention; p<0.05) [22].

A qualitative study conducted by Brumby and Smith (2009) showed the impact of the “Sustainable Farm Families Train the Trainer” model as a successful approach to mental health and wellness in farming populations [21]. Through this program, participants, who were service providers in the community, increased their knowledge around how mental health impacts farmers and their farm. It showed the value of engagement with farmers through a “farmer centred model of care” versus the traditional health model [21]. Women were more likely to access supports than men, and women experienced stress, in the context of their farm, differently than men [21]. Additionally, results showed that farmers maintained a “masculine” or “stoic” view of accessing mental health services, emphasizing the need for a farmer-based approach, grounded in the agricultural culture, to mental health services in agriculture [21].

A longitudinal cohort, the Australian Rural Mental Health Study, recruited participants from 2007–2009, to examine mental health outcomes and variables surrounding help-seeking for mental health issues, among farmers and non-farmers, across four time points (122). Results showed that farmers were significantly less likely than non-farmers to access services, regardless of the service accessibility within the community [28]. The authors discussed the importance of tailoring services to farmers to improve trust and improve service use.

A short report published in 2009 by Robinson and colleagues discussed the need for innovation through an initiative of the Centre for Rural and Remote Mental Health (Australia) aimed at enhancing existing models to better serve rural communities, including farmers [29]. Participating psychiatrists were surveyed to assess their level of comfort in serving farming populations by partnering with existing agencies that farmers accessed regularly (unrelated to mental health). Particularly, the authors discussed the role of partnering with farm financial advisors as a way to increase access to mental health services [29]. No data were presented in this short report.

Another short report, published in 2009, assessed the efficacy of a mental health support line for farmers in New South Wales who experienced severe draught [30]. This assessment used a mixed-methods approach, analyzing data collected by the Centre for Rural and Remote Mental health on a monthly basis, along with conducting qualitative interviews with phone line staff (n = 5). Regression analysis suggested that the number of calls was not significantly associated with the percentage of the state declared in drought. However, no data were presented and no overview of the approach to the qualitative data was discussed [30].

A qualitative study, published in 2009, explored mental health service providers’ perceptions, through oral histories, of how climate change has impacted the interdependency of political, environmental, economic, and mental health concerns in rural and remote communities that serve farmers [25].

The value of Mental Health First Aid (MHFA) training for Advisory and Extension agents working with farmers was evaluated in a pilot project conducted by Hossain et al in 2010 [27]. This study assessed the impact of MHFA training one-year post delivery. Using a mixed-methods approach, participants (n = 15) were surveyed and two focus groups were also conducted. Results showed “moderate” to “good” gains in understanding of mental health issues, “pathways to address” mental health issues, and a gain in confidence with respect to recognizing mental struggle and providing support to farmers to help them access appropriate services [27]. Participants rated MHFA as “moderately” to “quite” beneficial. The qualitative data were analyzed for themes; the specific approach to the qualitative analysis was not discussed [27].

In 2011, another Australian study provided an overview of using a community development approach to developing services, including mental health literacy training, in hopes of increasing service use by farming populations in New South Wales [23]. This region-based approach to service development allowed working groups (including industry groups, governmental agencies, and regional health and mental health service agencies) to adapt existing resources to meet the needs of their farming communities. Over the four-year project, over 3000 participants received mental health literacy training. The authors discussed the positive impact of this approach to development and delivery of mental health related services to rural areas [23]; however, no formal evaluation of the program was presented.

Farm-Link was another initiative in Australia (New South Wales) developed as a suicide prevention project [24], [31]; two studies were published. Funded from 2006–2011 through partnership between the Australian government and the University of Newcastle, this initiative aimed to promote prevention and early intervention for mental health issues, improve the care system, particularly in aiding in the coordination of multiple component parts that exist in rural areas to deliver mental health services [31]. Specifically, Farm-Link aimed to improve access to mental health services through improving linkages between existing agencies; both mental health agencies and other “trusted” agencies in the farming community (e.g. agricultural agencies and farm financial service agencies). Further, Farm-Link mapped these networks to report back to the agencies in order to facilitate relationship building and sharing of workload, and to bolster farmer engagement with available services without overlap [24], [31]. An independent evaluation was conducted on this project, using a mixed-methods approach [31]. The evaluation indicated that there was a large need for mental health resources across agencies, and that most agencies did not have the capacity to deal with these needs. Overall, all agencies increased the number of other agencies that they shared information with, but improvements to programs or mental health outcomes among program users were not tested statistically [31].

A research protocol for a clinical trial was published in 2016 by Kennedy et al [20]. This protocol was for a digital intervention aimed at reducing stigma around personal experiences with suicide among males who have lived-experience of suicide (e.g., bereaved, previous attempts, or ideation) living in Australian farming communities. This study proposed using a mixed-methods design, multi-level evaluation of a web-based intervention where participants would have access to educational materials, resource information, shared stories from peers, and a personal section for goal setting [20]. Quantitatively, perceived stigma was to be assessed using an adaptation of a validated scale, the Stigma of Suicide Scale (SOSS). Qualitatively, authors proposed to explore personal insights around stigma. This trial was registered with the Australian and New Zealand Clinical Trials Registry (ACTRN: ACTRN12416000289415). SOSS scores were to be assessed at the beginning of the trial and again, upon completion [20].

#### United States (n = 4)

The Farm Partners program in New York state was the first mental health intervention to be discussed in the literature, published in 1998 [32]. This program aimed to increase awareness and use of community resources by farm families in order to address the high levels of occupational stress. Results showed that over four years, this program helped to refer farmers to services for financial advice, health services, support groups, mental health professionals, social services, legal services, and more [32]. Within our search, we did not find a formal evaluation of this program’s effectiveness.

Another study explored the “Sowing the Seeds of Hope” behavioural health program, which had representation in Iowa, Kansas, Minnesota, Nebraska, North Dakota, South Dakota, and Wisconsin [33]. It highlighted the impact of creating services that were delivered by providers who have knowledge of agriculture to ensure that services are culturally acceptable, affordable, and accessible within the region they are meant to serve [33]. There was no published formal evaluation of this program returned in our search.

One experimental study sought to determine the effectiveness of music therapy aimed at decreasing depression, anxiety, and social isolation among Mexican farmworkers in the southern US [34]. The authors reported that music therapy had a “medium” but non-significant effect on decreasing depression scores (7).

In 2011, a cross-sectional study conducted through the National AgriAbility Project, highlighting the mental health services and resources that were available to farmers throughout this network, which included 21 United States Department of Agriculture-funded State and Regional Projects [35]. The most commonly provided mental health resources were speakers at workshops, with brochures, papers, or articles to share information about mental/behavioural health issues [35]. Within our search, we did not find a formal evaluation of the effectiveness of this program published.

#### Canada (n = 1)

In Canada, one qualitative study published in the year 2000 described the development and evaluation of a mental health service for Saskatchewan farmers intended to help farmers cope with stress [36]. Beginning with a qualitative needs-assessment, this study highlighted gaps in education (around stress management, anger management, self-esteem, and communication), support group formation and maintenance, clinical services, general information, and advocacy. Based on the results of this assessment, the author developed a program to address these needs within the health region, partnering with existing organizations to help deliver services [36]. This program included workshops on topics such as stress, anger and grief in the rural context, coping, and stress resiliency. The author emphasized the importance of trust and credibility as major factors to consider when developing mental health resources in farming communities. Once trust was established with farmers, she was able to deliver these services more effectively, and attendance improved [36]. A partial “evaluability assessment” was conducted in 1995; however, the author indicated that this evaluation was incomplete, leaving assessment of the program’s effectiveness reliant on anecdotal evidence.

#### United Kingdom (n = 1)

In the UK, an overview of an ongoing in-person support service delivered in farming communities by two nurses with farming backgrounds was published in 2000 [37]. This service aimed to address farming accidents, mental health, and occupational-related diseases. This review did not include any data and a follow-up report with findings was not identified.

#### India (n = 1)

In India, a mental health intervention was implemented in the Maharashtra region, which had experienced an increase in suicides [38]. This intervention recruited “social health activists” and trained them to use a tailored screening tool for depression among farmers. This tool was developed by psychiatrists in the region. The goal of the intervention was to detect depression among farmers and refer them for services, such as counselling and long-term treatment, more effectively [38]. No results or evaluation of this program was presented in this short report.

#### Africa (n = 1)

The study conducted in the Democratic Republic of Congo was unique in that the intervention the authors were evaluating was farming itself [39]. Specifically, this randomized control trial measured the impact of an intervention, Pigs for Peace, on individuals who were from regions greatly impacted by conflict. Approximately 50% of their participants identified as farmers. The study showed a statistically significant positive effect of having livestock as a moderator for good mental health, lowering overall impacts of depression and post-traumatic stress disorder [39].

## Discussion

This study systematically maps the literature examining mental health outcomes among farming populations, worldwide. The results of this review highlight gaps in research on farmer mental health. For example, the majority of research examining mental health outcomes focused on stress, suicide, and depression. These mental health outcomes were reported concerns in the specific regions in which they were studied, and thus may warrant further investigation globally. Further, mental health outcomes that have been less studied, including anxiety, burnout, and positive mental health outcomes, such as resilience, may warrant further investigation to determine the trends across nations.

Until recently, there have been very few studies of national scope that have examined mental health outcomes in North America beyond suicide, and these results have yet to be published in the academic literature (currently limited to conference publications) [40]. While small-scale studies contribute to localized knowledge, successful programming for farmer mental health has been supported with broader epidemiological data [22], [41]. A systematic mapping of existing research can help researchers avoid unnecessary overlap and can help identify knowledge gaps to inform the distribution of funds to where resources may be most effectual.

Beyond migrant farmworkers, other farming populations were not always explicitly identified within the literature. Overall, 36.1% of the cited literature did not specify which types of farming populations the authors were studying. While there is likely to be overlap between farming subpopulations regarding some aspects of mental health, there may be important differences to explore. For example, in a North American context, farmers are greatly impacted by changes in trade agreements, supply management, and governmental regulations that are vastly different and dependent on the specific farming commodity [42], [43]. Knowing which farming population is being examined lends more specificity to the work and can help researchers to elucidate these relationships more clearly.

### Mental health outcomes

Existing systematic reviews of farmer mental health are limited to one review of suicide [11]. Based on the findings of this review, it is warranted that researchers also systematically examine other outcomes; for example, depression among farming populations was measured in 109 (33.3%) of all studies included here. A limitation of this scoping review is that we did not aim to determine the number of studies that quantify the prevalence or incidence of depression; however, providing an adequate number of studies quantifying the prevalence of depression exists, conducting a systematic review and meta-analysis could provide valuable insights into establishing the global risk of experiencing depression among farmers. It could also allow for an exploration of heterogeneity among studies, which would help determine the degree of bias within and between studies. Additionally, this could provide a comprehensive analysis of the methodological rigour with which the studies were conducted. While there are many studies of depression throughout the literature, the way in which depression was investigated varied in study design, sample size, and measurement tools used. Assessing which tools (i.e. validated scales, physician diagnosis, self-report) have been used in farming populations can help inform future work and the development of standardized methodological guidelines could help make studies across the globe more comparable. For example, the Core Outcome Measurements in Effectiveness Trials (COMET) initiative has created a database of clinical studies that contribute to the development “core outcome sets (COS)” [44]. These COS inform the most appropriate measurement tools for clinical outcomes across many areas of health and wellness, to ensure data are comparable across trials. This could also serve to improve the epidemiological rigour of studying mental health outcomes in farming populations.

There were several studies that investigated “mental health” without operationalizing their definition of what this term meant within the study context. This trend has largely diminished over time, with more recent studies providing clear operational definitions that can be more easily interpreted.

With the exception of stress, depression, and suicide, other mental health outcomes were infrequently studied. Burnout, a more recent construct to be measured among farmers, was investigated in two studies [40], [45]. Given the association between burnout and employee retention generally [46], farmers experiencing burnout may have increased burdens beyond those of other occupations, given the nature of farming as an occupation, lifestyle, [45] and identity [47].

The vast majority of research focused on negative mental health outcomes (i.e. mental health pathology), to the near exclusion of positive mental health outcomes. Only one outcome identified in the present review focused on a positive outcome: resilience. While it is important to examine negative mental health outcomes, it could be argued that it is detrimental to do so to the exclusion of positive mental health outcomes like resilience, quality of life, and life satisfaction, which are outcomes commonly studied in the field of positive psychology. Positive psychology is “the scientific study of optimal human functioning [that] aims to discover and promote the factors that allow individuals and communities to thrive” [48]. Expansion of positive psychology research amongst farmers could help improve understanding on what helps enhance farmers’ well-being, and what helps some farmers thrive, even in the face of the significant stresses they face. In 2005, a literature review conducted by Fraser and colleagues concluded that more research is needed to explore resilience among farmers worldwide [10]. Give that resilience is positively associated with mental health [49], focusing research and resources on assessing how resilience can be taught and increased among farmers should be a priority for future work.

### Mental health services

To date, few studies have published formal evaluations of their mental health interventions/services, with all published evaluations coming from Australia. Globally, farming populations can benefit from building on this Australian knowledge, adapting services to fit other farming contexts. Formal evaluation of the impact of these services would also help to bolster existing literature and provide statistical evidence that can be used to prioritize farmer mental health research and services and lobby for permanent funding.

Throughout the studies that aimed to assess interventions, there were common findings indicating that farmers require services that are specifically tailored to their population needs and that are delivered by service providers that have a foundational understanding of the specific pressures associated with farming, and how this may impact the mental health of farmers. For example, studies conducted in Canada [50], Australia [21], and the US [33] reported that farmers are more willing to seek help for their mental health if they know the care provider has a knowledge base of agriculture. This may impact the trust between the farmer and mental health provider, which a systematic review of barriers to help-seeking for mental health issues in young people reported as essential for help-seeking both short- and long-term [51].

Gender differences were highlighted in an Australian study examining help-seeking among farmers [21]. Women were more willing to seek help for mental health concerns than men, and this gender association was further impacted by whether or not the mental health service provider had a knowledge base of farming and agriculture.

Hence, future mental health interventions that are developed for farming populations may benefit from considering both farm-specific needs and potential gender differences when designing new mental health prevention and intervention strategies. Furthermore, existing mental health services may benefit from educating service providers around farming practices, lifestyle, and realities in the communities that they serve, increasing their credibility within the farming community, and potentially increasing the likelihood that farmers will seek help for their mental health.

### Study limitations

While this review aimed to comprehensively map the existing peer-reviewed literature, it is possible that studies were not captured within the search. We aimed to reduce this risk by using comprehensive databases and checking reference lists of literature reviews that were identified in the study prior to full-text screening. As reference lists of reviews were examined prior to full-text screening, we are unable to identify the number of additional studies that were included as a result of reference checking. Further, this review excluded studies that did not explicitly state that they were examining a farming population. This may have led to the exclusion of studies labelling their population as “rural”, but included farmers. Lastly, studies that were unavailable in English, or that were part of a textbook or thesis were excluded, which may have excluded some geographical regions from the review.

## Conclusions

This scoping review provides a critical overview of the literature examining mental health outcomes in farming populations, globally. While some geographic regions had a substantial body of literature, knowledge gaps remain including the prevalence of mental health outcomes, how they are impacted by risk and protective factors, and which intervention strategies are most impactful in farming communities. The results of this scoping review can help guide researchers to identified gaps in research and services, leading to a more informed and focused approach to future work, and ultimately a more comprehensive understanding of mental health and wellness among farmers worldwide.

## Supporting information

S1 Appendix Data extraction form. (PDF)

S1 Table Reference list of included studies. (XLSX)

S1 Checklist PRISMA-ScR checklist. (PDF)
