# Supplementary material for: Research trends in farmers’ mental health: A scoping review of mental health outcomes and interventions among farming populations worldwide
Source: PLoS One. 2019 Dec 5;14(12):e0225661. doi: 10.1371/journal.pone.0225661 (PMC6894791; doi:10.1371/journal.pone.0225661)
Supplement: S1 Appendix — (PDF) [file pone.0225661.s001.pdf]

headers_table_report(2)

Question Text	Type	Question Header	Question Validation	Answer Text	Answer Header	Answer Validation
**Is the text available in English?**		Is the text available in English?		Yes	Yes	
				No	No	
**Based on the title and abstract, is the article relevant?**		Based on the title and abstract, is the article relevant?		Yes	Yes	
				No	No	
**What year was the study conducted?**		What year was the study conducted?				
**What year was the study published?**		What year was the study published?				
**What was the source of the study?**		What was the source of the study?		Journal	Journal	
				Conference Proceedings/Abstract	Conference Proceedings/Abstract	
				Government Report	Government Report	
				Website	Website	
				Thesis	Thesis	
				Editorial	Editorial	
				Textbook	Textbook	
				Other	Other	
**Which country/countries was the study conducted in?**		Which country/countries was the study conducted in?		Country/Countries	Country/Countries	
				If Canada, Which province	If Canada, Which province	
**Was this article:**		Was this article:		Primary research	Primary research	
				Review article	Review article	
				Editorial	Editorial	
				Secondary research	Secondary research	
**If secondary research, was it:**		If secondary research, was it:		Quantitative	Quantitative	
				Qualitative	Qualitative	
				Mixed Methods	Mixed Methods	
**If primary research, was it:**		If primary research, was it:		Mixed methods	Mixed methods	
				Quantitative	Quantitative	
				Qualitative	Qualitative	
**If quantitative (or mixed methods), was it:**		If quantitative (or mixed methods), was it:		Observational	Observational	
				Experimental	Experimental	
				Both	Both	
**If quantitative (or mixed methods), what was the study design?**		If quantitative (or mixed methods), what was the study design?		Cross-sectional	Cross-sectional	
				Cohort	Cohort	
				Case-control	Case-control	
				RCT	RCT	

**If observational, was it:**		If observational, was it:		Descriptive	Descriptive	
				Hypothesis testing	Hypothesis testing	
				Both	Both	
**If descriptive (or both), was it:**		If descriptive (or both), was it:		Evaluative: attitutes, stigma, etc	Evaluative: attitutes, stigma, etc	
				Estimating an outcome	Estimating an outcome	
				Development/modification of a diagnostic criteria	Development/modification of a diagnostic criteria	
**if it was hypothesis testing, (or both) was it:**		if it was hypothesis testing, (or both) was it:		Evaluative an intervention	Evaluative an intervention	
				Identifying risk factors	Identifying risk factors	
				Evaluation/validation of diagnostic criteria	Evaluation/validation of diagnostic criteria	
**If it was experimental/intervention, was it:**		If it was experimental/intervention, was it:		Drugs	Drugs	
				Behaviour modification	Behaviour modification	
				Psychotherapy	Psychotherapy	
				Health measures	Health measures	
				Other	Other	
**If qualitative (or mixed methods), was it:**		If qualitative (or mixed methods), was it:		Phenomenology	Phenomenology	
				Grounded theory	Grounded theory	
				Ethnographic	Ethnographic	
				Case study	Case study	
				Biography	Biography	
				Other	Other	
**Does the study describe a mental health service?**		Does the study describe a mental health service?		No	No	
				Yes	Yes	
**Who was included in the study population (check all that apply)?**		Who was included in the study population (check all that apply)?		Animal farmers	Animal farmers	
				Plant agriculture farmers	Plant agriculture farmers	
				Permanent farm workers	Permanent farm workers	
				Migrant farm workers	Migrant farm workers	
				Farm families	Farm families	
				Not specified	Not specified	
				Other:	Other:	
**If animal farmers, which commodity (check all that apply):**		If animal farmers, which commodity (check all that apply):		Swine	Swine	
				Beef	Beef	
				Dairy cattle	Dairy cattle	
				Small ruminants: meat or dairy	Small ruminants: meat or dairy	
				Poultry	Poultry	
				Aquaculture	Aquaculture	
				Other	Other	
**If plant agriculture, which commodity (check all that apply):**		If plant agriculture, which commodity (check all that apply):		Crops	Crops	
				Horticulture	Horticulture	
**What outcome(s) were measured (check all that apply):**		What outcome(s) were measured (check all that apply):		Suicide/suicide ideation/suicide attempt	Suicide/suicide ideation/suicide attempt	

				Depression	Depression	
				Anxiety	Anxiety	
				Stress/stressors	Stress/stressors	
				Mortality	Mortality	
				Burnout	Burnout	
				Resilience	Resilience	
				Not specified	Not specified	
				Other	Other	
**What year did the service become available?**		What year did the service become available?				
**Is the service still available?**		Is the service still available?		Yes	Yes	
				No	No	
**If the service is no longer available, why (check all that apply)?**		If the service is no longer available, why (check all that apply)?		Funding	Funding	
				Lack of use	Lack of use	
				Other resource issue	Other resource issue	
				Not stated	Not stated	
				Other	Other	
**Is the service informational or interventional?**		Is the service informational or interventional?		Informational	Informational	
				Interventional	Interventional	
				Both information and intervention	Both information and intervention	
				Other	Other	
**If informational, how was it delivered?**		If informational, how was it delivered?		Website	Website	
				Emailed directly to user	Emailed directly to user	
				Mail	Mail	
				In person	In person	
				Other	Other	
				Not Specified	Not Specified	
**How was the intervention delivered (check all that apply):**		How was the intervention delivered (check all that apply):		In person	In person	
				Online	Online	
				Telephone	Telephone	
				Other	Other	
**Who developed the service (check all that apply):**		Who developed the service (check all that apply):		Government	Government	
				Physicians	Physicians	
				Community members	Community members	
				Farmers	Farmers	
				Non-profit/NGO	Non-profit/NGO	
				Industry	Industry	
				Other	Other	
**If a governmental agency, was it (check all that apply):**		If a governmental agency, was it (check all that apply):		Local/municipal	Local/municipal	
				Provincial/state	Provincial/state	
				National	National	

**Who delivered the service (check all that apply):**		Who delivered the service (check all that apply):		Government	Government	
				Physicians	Physicians	
				Community members	Community members	
				Farmers	Farmers	
				Non-profit/NGO	Non-profit/NGO	
				Industry	Industry	
				Other	Other	
**If a governmental agency, was it (check all that apply):**		If a governmental agency, was it (check all that apply):		Local/municipal	Local/municipal	
				Provincial/state	Provincial/state	
				National	National	
**Who funds the service (check all that apply)?**		Who funds the service (check all that apply)?		Government	Government	
				Private: specify	Private: specify	
				Industry	Industry	
				Non-profit/NGO	Non-profit/NGO	
				Other	Other	
**If a governmental agency, was it (check all that apply):**		If a governmental agency, was it (check all that apply):		Local/municipal	Local/municipal	
				Provincial/state	Provincial/state	
				National	National	
**Is the service permanent?**		Is the service permanent?		Yes	Yes	
				No	No	
				Other	Other	
**Has the service been evaluated?**		Has the service been evaluated?		Yes	Yes	
				No	No	
